# Interleukin 6 in cerebrospinal fluid is a biomarker for delayed cerebral ischemia (DCI) related infarctions after aneurysmal subarachnoid hemorrhage

**DOI:** 10.1038/s41598-020-79586-3

**Published:** 2021-01-08

**Authors:** Sami Ridwan, Alexander Grote, Matthias Simon

**Affiliations:** 1 Department of Neurosurgery, Bethel Clinic, Bielefeld, Germany; 2 Department of Neurosurgery, Klinikum Ibbenbueren, Große Str. 41, 49477 Ibbenbueren, Germany

**Keywords:** Prognostic markers, Outcomes research

## Abstract

Interleukin 6 (IL-6) is a prominent proinflammatory cytokine and has been discussed as a potential biomarker for delayed cerebral ischemia (DCI) following aneurysmal subarachnoid hemorrhage. In the present study we have analyzed the time course of serum and cerebrospinal fluid (CSF) IL-6 levels in 82 patients with severe aneurysmal subarachnoid hemorrhage (SAH) requiring external ventricular drains in correlation to angiographic vasospasm, delayed cerebral ischemia, secondary infarctions and other clinical parameters. We observed much higher daily mean IL-6 levels (but also large interindividual variations) in the CSF than the serum of the patients with a peak between days 4 and 14 including a maximum on day 5 after SAH. Individual CSF peak levels correlated significantly with DCI (mean day 4–14 peak, DCI: 26,291 ± 24,159 pg/ml vs. no DCI: 16,184 ± 13,163 pg/ml; *P* = 0.023). Importantly, CSF IL-6 levels differed significantly between cases with DCI and infarctions and patients with DCI and no infarction (mean day 4–14 peak, DCI with infarction: 37,209 ± 26,951 pg/ml vs. DCI, no infarction: 15,123 ± 11,239 pg/ml; *P* = 0.003), while findings in the latter patient group were similar to cases with no vasospasm (mean day 4–14 peak, DCI, no infarction: 15,123 ± 11,239 vs. no DCI: 15,840 ± 12,979; *P* = 0.873). Together, these data support a potential role for elevated CSF IL-6 levels as a biomarker for DCI with infarction rather than for DCI in general. This fits well with a growing body of evidence linking neuroinflammation to ischemia and infarction, but (together with the large interindividual variations observed) limits the diagnostic usefulness of CSF IL-6 levels in SAH patients.

## Introduction

Spontaneous aneurysmal subarachnoid hemorrhage (SAH) is a common cerebrovascular disease and neurosurgical emergency associated with serious complications and a high mortality rate requiring timely aneurysm occlusion and constant neurosurgical and critical care observation during the acute phase^1–8^. A major complication following SAH is the occurrence of delayed cerebral ischemia (DCI). The term is commonly used to describe a clinical syndrome consisting of secondary neurological impairment often responsive to induced hypertension and not attributable to aneurysm occlusion, (treatment of) hydrocephalus, seizures, septic complications or other competing causes. A very sizable number of patients with DCI develop cerebral infarcts^5,9–11^. Secondary cerebral infarction is a major cause of poor clinical outcome or even death following SAH^3,4,12^. The term vasospasm (VS) was originally coined to describe the somewhat mechanistic concept of major artery narrowing causing neurological deterioration due to territorial perfusion deficits which may or may not result in infarction. However, the correlation between VS, i.e. arterial narrowing demonstrated by angiography and DCI and infarction is not perfect, and a more recent view of the pathophysiology of DCI will acknowledge a role for additional factors such as involvement of small arteries, thromboembolism, spreading depressions and neuroinflammation^5,13–20^. While DCI is primarily seen in patients with aneurysmal SAH, it may occur occasionally in cases in which no aneurysm can be demonstrated and even complicate the treatment of completely unrelated conditions such as traumatic brain injury, brain tumor and CNS infections^21–24^.

Of note, defining and diagnosing DCI in the clinical context is not a trivial matter. Most would probably use a combination of clinical (see above, secondary neurological worsening after exclusion of competing cause, secondary neurological deficits responsive to induced hypertension) and transcranial Doppler sonography and/or imaging findings (VS shown by DSA or CTA, regional perfusion deficits and infarctions documented by CT or MR studies)^5,22,25,26^. The occurrence of imaging proven secondary infarctions might well be the most robust and relevant criterion in the context of a clinical study^9–11^. However, it can only be used in a post-hoc setting i.e. it is not suitable for intervention studies since it does not account for successful treatment. Therefore, identification of a diagnostic biomarker would be a major step forward in the management of DCI.

Modern intensive care facilities allow for detailed monitoring of numerous clinical, technical as well as laboratory variables which have been associated with the risk for DCI and other SAH related complications and patient outcome^3,6–8,27^. Interleukin (IL)-6 in the cerebrospinal fluid (CSF) and serum have been discussed in some detail as possible biomarkers of DCI. IL-6 is commonly regarded as a prominent mediator of the inflammatory response following SAH. Several investigators have therefore analyzed IL-6 levels in SAH patients, and some studies describe a correlation between elevated IL-6 levels and DCI^5,19,20,28–33^. IL-6 levels may correlate with the patients’ health related quality of life (HRQoL)^34^.

At the authors’ institution, determination of serum and CSF IL-6 is performed as part of the routine monitoring and management of intensive care patients with aneurysmal SAH which made serial CSF and serum IL-6 determinations available from a relatively large patient series. In view of the possible correlation between increased IL-6 levels and DCI just outlined, we analyzed these data in order to evaluate IL-6 as a possible diagnostic (and prognostic) biomarker for DCI. The identification of a DCI biomarker might be particularly relevant for patients with severe SAH who are often sedated and ventilated and therefore cannot be monitored by serial neurological exams.

## Patients and methods

### Patients; treatment and outcome data

The hospital’s database was searched to identify patients treated for SAH in the Department of Neurosurgery of the Bethel Clinic in Bielefeld, Germany from February 2016 through February 2019. Cases without aneurysms and patients with AVM associated aneurysms were excluded. In 82 consecutive cases requiring external ventricular drainage serial CSF IL-6 levels (≥ 6 samples; ≥ 3 samples between days 4 to 14) could be made available. These patients were included in the final analysis.

Pertinent demographic (age, sex), laboratory, radiological and treatment data were extracted from the patients’ medical records including time of bleeding, World Federation of Neurosurgical Societies (WFNS) grade, Fisher grade^35^, location of the aneurysm, time of occlusion and occlusion technique (clipping vs. coiling). We also documented the specifics of all additional surgical or neurointerventional measures (e.g., decompressive hemicraniectomy, placement of external ventricular drains, VP shunt surgery, intraarterial spasmolysis with nimodipine) and medical complications (e.g., meningitis; including microbiology and routine CSF analysis findings). The modified Rankin Scale (mRS) was used to describe the patients’ clinical outcome at discharge. A good clinical outcome was defined as mRS 0–2 in accordance with the literature^36–38^. All clinical, radiological and laboratory data were analyzed retrospectively. No study-specific investigations or tests were performed. In particular, serum and CSF IL-6 levels were obtained as part of the routine monitoring of intensive care patients with aneurysmal SAH. The study was approved by the responsible ethics committee (Ethikkommission der Ärztekammer Westfalen-Lippe und der Westfälischen Wilhelms-Universität Münster, Az 2019–202-f-S). All procedures involving human participants were in accordance with the ethical standards of the institutional and all national research committees and with the 1964 Helsinki Declaration and its later amendments or comparable ethical standards. Individualized informed consent was obtained from all patients or their legal guardians for hospital treatment including all diagnostic and treatment modalities as well as for statistical analyses of all clinical and laboratory data.

### Definition of DCI (delayed cerebral ischemia) and VS (vasospasm)

All medical records and radiological studies were reviewed and assessed for the occurrence and the time of onset of clinical symptoms of DCI and cerebral infarctions. Cerebral infarctions were classified as aneurysm treatment-related (i.e., perforator ischemia after anterior communicating aneurysm clipping or embolic infarcts after aneurysm coiling; documented by CT < 24–48 h. following aneurysm occlusion) or secondary infarction (new finding shown by CT/MRI > 48 h. after aneurysm treatment and not seen on the < 24–48 h. scan, which could not be explained by the aneurysm occlusion or any other neurosurgical or neuroradiological procedure). All patients had a CT or MR scan within 48 h. of aneurysm occlusion.

Per routine all SAH patients had daily TCD examinations from day ≤ 3 to 14 (if awake and without symptoms) or 21 (if sedated). Cases with a clinical diagnosis of DCI had TCD examinations until symptoms resolved and at least until day 21. CT angiography/perfusion studies were obtained as indicated in order to identify cases with angiographic VS and perfusion deficits. Patients had conventional angiographies for aneurysm diagnosis (all cases), for aneurysm coiling, for clip control (per routine on day 10–14) and for salvage spasmolysis (see Results).

For the purpose of this study, DCI was defined as imaging evidence of secondary infarctions unrelated to the aneurysm occlusion procedure or other interventions; and/or secondary neurological impairment after exclusion of competing causes, responsive to induced hypertension with or without secondary infarcts. VS was defined by pathognomonic transcranial Doppler findings (mean MCA or ACA flow > 120 cm/s and Lindegaard index > 3) and/or angiographic vasospasm (conventional angiography or CT angiography/perfusion study).

### Interleukin 6 analysis

IL-6 levels in CSF and serum [pg/ml] were obtained per routine (Electrochemiluminescence immunoassay; ELECSYS IL6, Roche Diagnostics GmbH, Mannheim, Germany) directly after a CSF drain was placed and daily thereafter until the drain was removed. VS is most often seen between days 4 and 14 following SAH, and several groups investigating a possible link between IL-6 levels and vasospasm have hence focused on day 4 to 14 IL-6 findings^5,28,29,34,39^. For the purposes of our analysis we have therefore studied day 0–3 and 4–14 CSF IL-6 peak values separately in addition to overall CSF and serum peaks (day 0–28).

### Statistical analysis

Statistical analysis was performed using a commercially available software (IBM SPSS Statistics for Windows, Version 25.0, IBM Corp., Armonk, NY). Standard procedures (Fisher exact test, chi-square test, trend tests, Student t-test, ANOVA) were used for univariate analyses as indicated. IL-6 levels were studied using mean and maximum values, and logistic regression analysis. Receiver operating characteristics (ROC) analysis was used to assess the potential of CSF IL-6 levels as a diagnostic marker.

## Results

### Patient cohort and aneurysm treatment

We enrolled 82 cases with aneurysmal subarachnoid hemorrhage which required CSF drainage in this analysis. The majority of the external ventricular drains (EVD) were placed within 24 h. of admission (N = 76/82; 92.7%). Table 1 lists patient, treatment and outcome data. The patient cohort consisted of 51 women and 31 men. Median age was 58 years (range 26–90 yrs.).

**Table 1:** Patient demographics, treatment and outcome data.

Age (yrs., range, mean ± SD)	26–90, 59.0 ± 14.5	
Sex (female/male)	51/31	62.2%/37.8%
**WFNS grade**		
1	18	22.0%
2	20	24.4%
3	7	8.5%
4	24	29.3%
5	13	15.9%
**Fisher grade**		
1 & 2	0	0
3	45	54.9%
4	37	45.1%
**Aneurysm location**		
AcomA	31	33.8%
PCA	2	2.4%
ICA (w/o PcomA & AchoA)	5	6.1%
AchoA	3	3.7%
PcomA	14	17.1%
MCA	21	25.6%
VA/PICA	4	4.9%
BA	2	2.4%
**Aneurysm treatment**		
Clipping	46	56.1%
Coiling	34	41.5%
Conservative	2	2.4%
**Hemicraniectomy**		
Primary	21	25.6%
Secondary	5	6.1%
**Time to admission**		
≤ 24 h	76	92.7%
> 24 h	6	7.3%
**Time to aneurysm occlusion**^1^		
≤ 24 h	61	76.3%
24–72 h	11	13.8%
> 72 h	8	10.0%
Meningitis^2^	8	9.8%
CSF shunt	26	31.7%
**Outcome at discharge**		
mRS 0–2	23	28.0%
mRS 3–6	59	72.0%

^1^Excluding 2 cases managed conservatively.

^2^Culture-positive meningitis.

*AchoA* anterior choroidal artery, *AcomA* anterior communicating artery, *BA* basilar artery, *CSF* cerebrospinal fluid, *h*. hours, *ICA* internal carotid artery, *MCA* middle cerebral artery, *mRS* modified Rankin scale, *PCA* posterior cerebral artery, *PcomA* posterior communicating artery, *PICA* posterior inferior cerebellar artery, *SD* standard deviation, *VA* vertebral artery, *WFNS* World Federation of Neurosurgical Societies, *yrs*. years.

Thirty-eight patients (46.3%) were admitted directly to our emergency room, while 44 cases were referred from outside hospitals with no neurosurgical department. Six patients were admitted > 24 h. (range 2–9 days) after SAH. Clinical presentation (WFNS grades), initial CT findings (Fisher grades), aneurysm location and treatment are detailed in Table 1. The majority of patients (63/82, 76.8%) had their aneurysm treated within 24 h. Two cases (2.4%) were managed conservatively because of their poor clinical condition. A hemicraniectomy or posterior fossa decompression (one patient) was performed in 21 cases (25.6%) as part of their primary (≤ 24 h.) treatment (clipping: 16, coiling: 5). Five patients (6.1%) had a hemicraniectomy > 24 h. for otherwise uncontrollable intracranial hypertension.

### DCI, infarctions, VS monitoring and clinical outcomes

DCI as defined in the Methods section was eventually diagnosed in 34 (41.5%) cases. Of note, this includes 22 (26.8%) patients with DCI related delayed infarctions as evidenced by CT scanning. DCI was diagnosed 8.3 ± 3.4 days following SAH.

All patients with DCI and/or VS were treated with induced hypertension (MAD > 100 mmHg). Salvage spasmolysis with intraarterial nimodipine was performed in 13 vasospastic patients. Permanent CSF shunting was required in 26 cases (31.7%). Favorable outcomes (= mRS 0–2) at discharge were seen in 23 (28.0%) of our patients. mRS 0–3 outcomes at discharge were observed in 34 (41.5%) cases.

### Interleukin 6 CSF levels predict secondary infarctions rather than DCI

**Figure 1:**
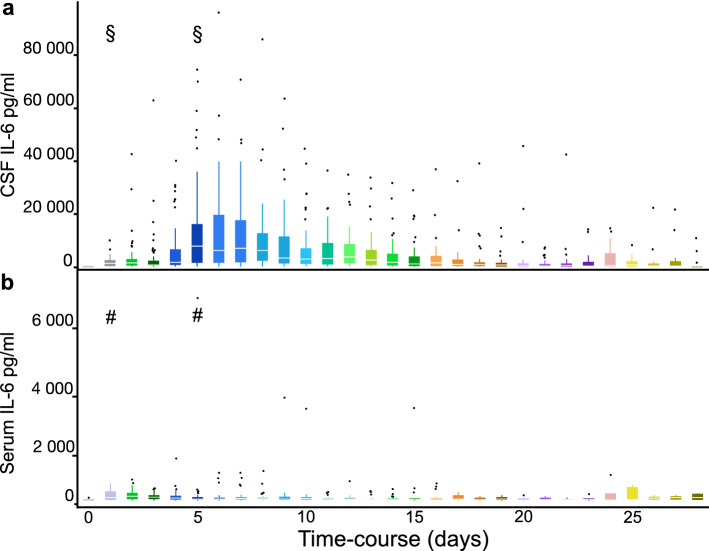
Interleukin 6 CSF and serum levels following aneurysmal SAH. CSF and blood serum IL-6 levels from day 0 to day 28 are depicted as box plots. The median is marked by the white notch within the quartiles. Outliers are plotted as individual points. IL-6 CSF levels (**a**) were distinctly higher than serum levels (**b**) throughout the study period. There was a significant increase over the first few days and the peak median CSF IL-6 value was observed at day 5 after subarachnoid hemorrhage (§ in 1a; day 5 vs. day 1, *P* < 0.01). No corresponding peak was seen for serum IL-6 levels (# in 1b; day 5 vs. day 1, *P* = 0.14).

Serial IL-6 determinations allowed for an assessment of IL-6 levels over time following SAH. Serum IL-6 levels were available in 76/82 (92.7%) cases. Daily IL-6 CSF and serum levels are shown in Fig. 1. On average, CSF IL-6 levels were much higher than serum levels. Also, CSF levels appeared to vary systematically over time with a peak between days 4 and 14 including a maximum on day 5 after SAH, however, there was very significant interindividual variation. No such systematic changes were seen for serum IL-6 levels.

Next, we addressed the principal question of our study, i.e., a possible association between CSF IL-6 levels and DCI. Indeed, individual CSF IL-6 day 4–14 peak levels were found to correlate significantly with DCI as demonstrated in Table 2. Baseline CSF IL-6 levels following SAH have also been suggested as predictors of DCI^19,40^. We could not confirm such findings when studying peak CSF IL-6 day 0–3 levels (Table 2). We also investigated the ratio between the day 4–14 and day 0–3 peak values as a measure of the relative CSF IL-6 increase. No significant associations were observed. There were no correlations between individual serum IL-6 peak levels and DCI (Table 2). Same day CSF and serum day 4–14 levels were available in 70 (85.4%) cases which allowed for a calculation of the ratio between CSF and serum findings. We observed no correlations with DCI. Importantly, we also found no correlations between IL-6 levels and VS (Table 2).

**Table 2:** Individual interleukin-6 peak levels in correlation with Vasospasm and DCI.

	Vasospasm (VS)			DCI		
	Mean ± SD (pg/ml)		Logistic regression (per 1000 pg/ml or per 1 for ratios)	Mean ± SD (pg/ml)		Logistic regression (per 1000 pg/ml or per 1 for ratios)
	Yes	No	OR (95% CI)	Yes	No	OR (95% CI)
CSF IL-6 peak	24,782 ± 21,738	21,956 ± 18,551	1.007 (0.985–1.030)	27,932 ± 23,095	19,495 ± 16,723	1.022 (0.998–1.046)
	*P* = 0.540		*P* = 0.536	*P* = 0.061		*P* = 0.068
CSF IL-6 day 0–3 peak	2536 ± 3358	6467 ± 12,102	0.904 (0.798–1.024)	2926 ± 3566	5577 ± 11,422	0.949 (0.867–1.039)
	*P* = 0.083*		*P* = 0.112*	*P* = 0.186*		*P* = 0.255*
CSF IL-6 day 4–14 peak	22,386 ± 21,945	19,319 ± 16,639	1.008 (0.985–1.032)	26,291 ± 24,159	16,184 ± 13,163	1.029 (1.003–1.056)
	*P* = 0.494		*P* = 0.490	*P* = 0.023		*P* = 0.028
CSF IL-6 day 4–14 peak/ day 0–3 peak	19.3 ± 27.7	13.2 ± 18.7	1.012 (0.990–1.034	18.6 ± 25.8	14.7 ± 22.6	1.007 (0.987–1.027)
	*P* = 0.293*		*P* = 0.298*	*P* = 0.508*		*P* = 0.505*
Serum IL-6 peak (pg/ml, mean ± SD)	466 ± 722	462 ± 1295	1.004 (0.637–1.583)	465 ± 1203	464 ± 771	1.001 (0.638–1.571)
	*P* = 0.986**		*P* = 0.986**	*P* = 0.997**		*P* = 0.997**
Serum IL-6 day 0–3 peak (pg/ml, mean ± SD)	206 ± 209	165 ± 188	2.970 (0.150–58.839)	174 ± 177	198 ± 217	0.529 (0.027–10.265)
	*P* = 0.483***		*P* = 0.475***	*P* = 0.681***		*P* = 0.674^#^
Serum IL-6 day 4–14 peak (pg/ml, mean ± SD)	406 ± 752	316 ± 1263	1.102 (0.660–1.841)	430 ± 1280	308 ± 634	1.138 (0.691–1.876)
	*P* = 0.711^#^		*P* = 0.709^#^	*P* = 0.610^#^		*P* = 0.611***
CSF IL-6 day 4–14 / same day serum IL-6 peak (mean ± SD)	484 ± 503	717 ± 933	1.000 (0.999–1.000)	634 ± 794	536 ± 657	1.000 (1.000–1.001)
	*P* = 0.223^#^		*P* = 0.198^#^	*P* = 0.576^#^		*P* = 0.572^#^

*, **, ***, ^#^: N = 71, 76, 47, 70.

*CSF* cerebrospinal fluid; *CI* confidence interval; *DCI* delayed cerebral ischemia, *IL-6* interleukin-6; *OR* odds ratio; *SD* standard deviation.

CSF IL-6 levels have also been discussed as potential markers of meningitis and ventriculitis^33^. In our cohort, the mean overall and day 4–14 CSF IL-6 peaks were not higher in patients with versus without culture-positive meningitis as shown in Table 3. In order to further exclude the possibility that elevation of IL-6 levels due to bacterial meningitis might obscure the association between IL-6 levels and DCI we also studied mean IL-6 levels as potential predictors of DCI after exclusion of cases with culture-positive meningitis. Similar to the findings in the overall cohort, peak overall CSF IL-6 and day 4–14 levels correlated strongly with DCI. DCI versus no DCI, overall peaks: 31,019 ± 24,597 versus 18,169 ± 15,382, *P* = 0.012 and day 4–14 peaks: 30,307 ± 25,237 versus 15,840 ± 12,979, *P* = 0.005. Since diagnosing bacterial meningitis solely by positive microbiological cultures certainly underestimates the true incidence of this complication^33^, we identified all patients with CSF pleocytosis (> 100 × 10^6^/l) and clinical signs compatible with an infection. We will acknowledge that also many cases with aseptic meningitis will meet these criteria. After exclusion of these patients (N = 31) from the analysis we found again very significant correlations between IL-6 peak levels and DCI (DCI vs. no DCI, overall peaks: 30,438 ± 24,033 vs. 18,774 ± 15,950, *P* = 0.017 and day 4–14 peaks: 28,645 ± 25,375 vs. 15,780 ± 12,562, *P* = 0.009).

**Table 3:** Correlations between individual CSF interleukin-6 peak levels and clinical parameters.

	CSF IL-6 peak (pg/ml, mean ± SD)	*P*	CSF IL-6 day 4–14 peak (pg/ml, mean ± SD)	*P*
**Age**				
≤ 58 yrs	22,400 ± 20,786		21,184 ± 20,966	
> 58 yrs	24,821 ± 20,201	0.594	21,044 ± 18,957	0.975
**Sex**				
Female	21,742 ± 18,748		19,627 ± 19,137	
Male	26,683 ± 22,863	0.290	23,560 ± 21,095	0.388
**WFNS**				
1	20,792 ± 22,198		18,795 ± 21,832	
2	20,378 ± 13,006		17,624 ± 14,361	
3	26,209 ± 25,019		22,000 ± 23,651	
4	21,257 ± 18,954		18,309 ± 15,409	
5	35,430 ± 25,444	0.229	34,396 ± 26,287	0.124
**Fisher grade**				
3	19,757 ± 17,099		17,492 ± 16,759	
4	28,297 ± 23,202	0.059	25,519 ± 22,542	0.068
**Location**				
Ant. circulation	22,642 ± 20,114		20,144 ± 19,389	
Post. circulation	35,881 ± 21,946	0.127	33,402 ± 23,610	0.116
**Aneurysm treatment**				
Clipping	21,351 ± 19,596		18,453 ± 17,963	
Coiling	25,507 ± 18,817	0.343	23,407 ± 19,322	0.241
**Hemicraniectomy**				
Yes	23,857 ± 23,962		19,804 ± 21,832	
No	23,495 ± 18,766	0.941	21,722 ± 19,058	0.687
**Postclipping/coiling infarction**				
Yes	25,494 ± 17,498		22,546 ± 15,468	
No	22,276 ± 22,320	0.485	20,100 ± 22,571	0.586
**Meningitis**^1^				
Yes	22,542 ± 17,968		12,028 ± 10,518	
No	23,726 ± 20,758	0.877	22,096 ± 20,424	0.175
**CSF Shunt**				
Yes	21,369 ± 13,051		17,401 ± 12,230	
No	24,651 ± 23,071	0.415	22,838 ± 22,440	0.161
**Outcome at discharge**				
mRS 0–2	14,940 ± 11,488		13,742 ± 11,364	
mRS 3–6	26,990 ± 22,136	0.002	23,988 ± 21,727	0.007

^1^Culture-positive meningitis.

*CSF* cerebrospinal fluid, *SD* standard deviation, *WFNS* World Federation of Neurosurgical Societies, *yrs*. years.

The literature contains somewhat conflicting data with respect to an association between elevated IL-6 levels and DCI. We hypothesized that this might reflect to some degree the different criteria used to define DCI. Imaging evidence of secondary infarctions is a strict but also probably the most robust and relevant criterion with respect to patient outcome^9,10^. We therefore analyzed CSF IL-6 overall and day 4–14 levels separately in patients with secondary infarctions (Table 4). Importantly, both overall as well as day 4–14 levels differed very significantly between cases with infarcts and patients with DCI but no infarction (*P* = 0.005 and *P* = 0.003). Indeed, we observed very similar CSF IL-6 levels in these latter patients when compared to cases without DCI. Hence our data point to an association specifically between high CSF IL-6 levels and vasospastic infarctions (i.e., DCI not responsive to treatment).

**Table 4:** Individual interleukin-6 peak levels in patients with and without DCI and/or infarction.

	No DCI	DCI w/o infarction	DCI with infarction
CSF IL-6 peak (pg/ml, mean ± SD)	18,169 ± 15,382	17,307 ± 9497	37,252 ± 26,912
		*P* = 0.005 for DCI with versus DCI w/o infarction	
	*P* = 0.867 for DCI w/o infarction versus no DCI		
CSF IL-6 day 4–14 peak (pg/ml, mean ± SD)	15,840 ± 12,979	15,123 ± 11,239	37,209 ± 26,951
		*P* = 0.003 for DCI with versus DCI w/o infarction	
	*P* = 0.873 for DCI w/o infarction versus no DCI		

*CSF* cerebrospinal fluid, *DCI* delayed cerebral ischemia, *SD* standard deviation, *w/o* without.

**Figure 2:**
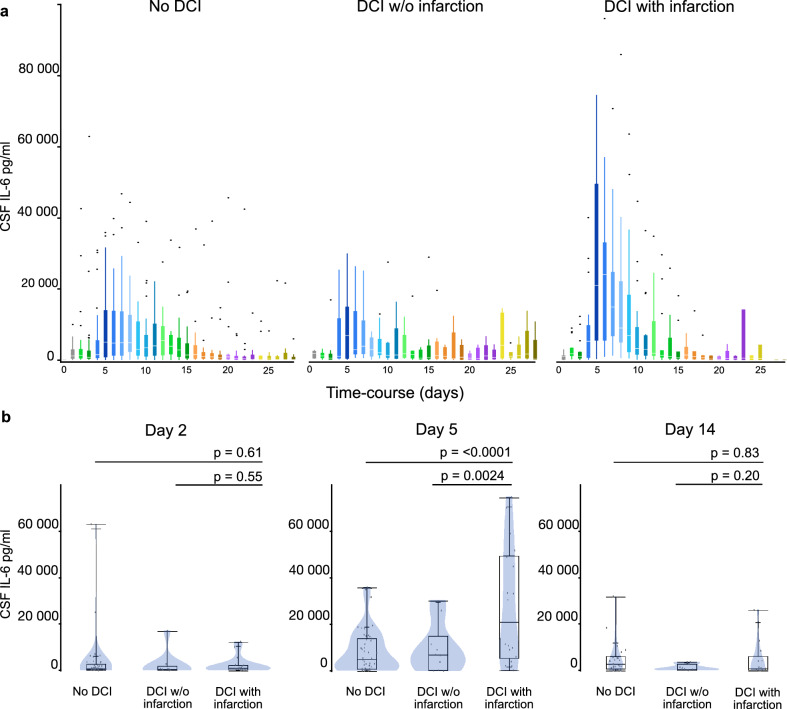
Interleukin 6 CSF levels in cases with no DCI, with DCI and no infarction, and in patients with DCI and infarction. (**a**) In all three patient groups a significant increase of CSF IL-6 levels was seen in the first week after SAH with a peak on day 5 (day 1 vs. day 5; no DCI *P* = 0.039, DCI w/o infarction *P* = 0.003, DCI with infarction *P* < 0.001). (**b**) Boxplots show the inter-group comparison for days 2, 5 and 14. While median CSF IL-6 levels did not differ significantly on day 2 (DCI with infarction vs. no DCI and ~ vs. DCI w/o infarction: *P* = 0.61 and *P* = 0.55), there were significant differences between median CSF IL-6 levels in the DCI with infarction group and both other groups (no DCI and DCI w/o infarction: *P* < 0.0001 and *P* = 0.0024) on day 5. This effect persists for several days (not shown) and is no longer seen on day 14 (DCI with infarction vs. no DCI and ~ vs. DCI w/o infarction: *P* = 0.83 and *P* = 0.20).

As a corollary, Fig. 2 shows daily CSF IL-6 values in patients with DCI and infarcts, with DCI and no infarcts, and cases with no DCI at all. Daily CSF IL-6 values peaked in all three patient groups on day 5 following SAH. These peaks differed significantly between DCI cases with and without (*P* = 0.0024). There was no significant difference between cases with DCI but no infarcts and cases without DCI (Fig. 2).

In order to assess the potential of CSF IL-6 determinations as a diagnostic marker for DCI and infarction we performed ROC analyses. Significant results were obtained, however, likely due to the relatively high interindividual variation the diagnostic potential of CSF IL-6 determinations is probably very limited. The optimal IL-6 cut-offs defined by the Youden index together with their associated sensitivity and specificity figures are shown in Table 5.

**Table 5:** Receiver operating characteristics (ROC) analysis.

	CSF IL-6 peak		CSF IL-6 day 4–14 peak	
	DCI	Infarction	DCI	Infarction
AUC (95% CI)	0.657 (0.531–0.783)	0.718 (0.579–0.856)	0.658 (0.530–0.787)	0.747 (0.615–0.879)
	*P* = 0.021	*P* = 0.003	*P* = 0.020	*P* = 0.001
**Optimal cut-off**	25,138	44,558	23,824	44,558
Sensitivity	56%	46%	56%	46%
Specificity	74%	94%	79%	98%

*AUC* area under the curve, *CI* confidence interval, *CSF* cerebrospinal fluid, *DCI* delayed cerebral ischemia/infarction.

### Interleukin 6 CSF levels as a biomarker for patient outcome after severe SAH

IL-6 is a proinflammatory cytokine and neuroinflammation may be an important pathomechanism underlying and modifying not only vasospastic ischemia, but also other aspects of SAH. We therefore explored our dataset also for correlations between CSF IL-6 levels and characteristics of the clinical course and treatment of SAH other than DCI (Table 3). The only significant association observed was with patient outcome. No patient with a CSF IL-6 day 4–14 peak > 35,000 pg/ml was found to have a mRS 0–2 (good) outcome. There was a trend (*P* < 0.1) towards higher IL-6 levels in Fisher grade 4 versus 3 bleedings and (albeit not statistically significantly) increased CSF IL-6 levels were seen in WFNS grade 5 SAHs. These latter findings point to some association between IL-6 levels and the initial severity of the bleed.

## Discussion

DCI and delayed infarcts are major causes of morbidity and mortality following SAH. The pathophysiology of DCI and secondary infarction remains unclear, but the inflammatory response elicited by a cerebral hemorrhage may play a significant pathogenetic role. This has prompted several groups to investigate IL-6 which is one of the more prominent proinflammatory cytokines as a biomarker for DCI^5,28,30–32,34,41,42^. The present study has two principal findings. Firstly, we provide data that confirm a correlation between elevated CSF IL-6 levels and DCI. Secondly, our analysis suggests that this correlation chiefly reflects high CSF IL-6 levels in patients who develop secondary infarctions.

A number of studies have investigated a potential correlation between elevated IL-6 levels and DCI. Most groups have published positive findings^5,29,33,40,41^. However, the respective associations were often weak and the wide range of actually measured values appeared to hamper its diagnostic usefulness. Our study confirms these earlier findings including the considerable interindividual variation of actual measurements. CSF rather than serum, and intermediate (day 4 to 14) rather than early (day 0–3) IL-6 levels seem to correlate with DCI. Absolute levels are most important and not the relative increase over time or the ratio between CSF and serum determinations. Our data do not necessarily contradict studies attributing some diagnostic value to these latter parameters. It is quite possible that the retrospective character of our analysis precluded the delineation of more subtle associations.

CNS infections might also result in elevated CSF IL-6 levels^33^. Lenski et al. have recently explored the usefulness of CSF IL-6 in differentiating between DCI and meningitis^33^. We found little evidence that elevated CSF IL-6 levels in our cohort reflect CNS infections. Of note, Lenski and co-workers used CSF IL-6 levels at the time of first diagnosis of DCI or meningitis rather than peak values over several days. While their approach may have been more sensitive than ours if one assumes that CSF IL-6 levels rapidly increase and decrease with the clinical course of meningitis it may have limitations with respect to patients with both, DCI and CNS infections.

It is quite possible that some of the discrepancies between the various studies investigating IL-6 in SAH simply reflect the problem of properly defining and diagnosing VS and DCI. Very intriguingly, we found high CSF IL-6 levels predominantly in cases with secondary (vasospastic) infarctions rather than patients with clinical or apparative findings thought to be pathognomonic for DCI. Our findings fit very well with a substantial body of data linking IL-6 to ischemic stroke. Indeed, IL-6 levels have been prominently discussed as potential biomarkers for ischemic stroke risk, outcome after stroke and even infarct size^43–45^. Most investigators have analyzed serum but a few also CSF IL-6 levels^28,30,32,34,41,42^. Importantly, these data underline the role of neuroinflammation in the pathophysiology of DCI. A focus on neuroinflammation will likely result in very different concepts of DCI prevention and treatment than the current emphasis on imaging-based diagnosis and angiographic treatment (i.e., intraarterial spasmolysis).

As pointed out, our data suggest that high CSF IL-6 levels will not identify patients who benefit from DCI treatment but rather those who will ultimately develop cerebral infarcts. This explains why CSF IL-6 levels were higher in patients with an adverse outcome in our study, since the occurrence of secondary infarcts is a major negative outcome predictor^9,11,46^. Very similar findings have also been reported by other groups^5,28,31,32,34^. However, possible associations of IL-6 levels with the severity of the bleed and other outcome relevant parameters also warrant consideration. Therefore, CSF IL-6 might have potential as a prognostic rather than as a diagnostic biomarker in SAH. We were unable to confirm an association with shunt-dependency as reported by others^30,39^.

We will readily acknowledge several important limitations of our study. Of note, we conducted a retrospective analysis of a dataset which was not primarily obtained with the intention to monitor and diagnose vasospasm. Similar to some prospective studies we allowed for the inclusion of patients who were not assayed daily^29,40^. We did not analyze other markers of (neuro)inflammation. However, the pertinent literature provides little evidence pointing to their relevance in SAH patients^14,20,29^. The exclusion of cases managed without ventricular drains might have introduced some selection bias even though our cohort appears representative, since the distribution of various clinical parameters such as WFNS grade, aneurysm location, incidence of meningitis and shunt dependency, and outcome is roughly similar to published series^28,31,32,34,41^.

## Conclusion

In summary, we observed a strong correlation between elevated CSF IL-6 levels and secondary vasospastic infarcts in a sizable series of SAH patients. CSF IL-6 is a prominent proinflammatory cytokine and therefore our study adds to the growing body of data that links neuroinflammation to DCI. However, CSF IL-6 levels were not significantly elevated in patients with DCI responsive to treatment (i.e., who did not develop infarcts). This and the wide range of measured values limit the usefulness of CSF IL-6 as a biomarker for the diagnosis of DCI.
